# Incidence and outcomes of neuromuscular immune-related adverse events in head and neck cancer patients treated with checkpoint inhibitors

**DOI:** 10.1038/s41598-025-31483-3

**Published:** 2025-12-05

**Authors:** Stefanie Glaubitz, Jana Zschüntzsch, Andreas Sakkas, Mario Scheurer, Frank Wilde, Alexander Schramm, Marcel Ebeling

**Affiliations:** 1 Department of Neurology, University Medical Center Göttingen, Robert-Koch-Straße 40, 37075 Göttingen, Germany; 2 Department of Oral and Plastic Maxillofacial Surgery, Military Hospital Ulm, Academic Hospital of the University of Ulm, Oberer Eselsberg 40, 89081 Ulm, Germany; 3 Department of Oral and Plastic Maxillofacial Surgery, University Hospital Ulm, Albert-Einstein-Allee 10, 89081 Ulm, Germany

**Keywords:** Head and neck cancer, Myasthenia gravis, Myositis, Neuritis, Immune checkpoint inhibitor, Cancer, Diseases, Immunology, Neurology, Oncology

## Abstract

Immune checkpoint inhibitors (ICIs) are a transformative therapy for many cancers, including head and neck cancer (HNC). However, their unique mechanism can trigger immune-related adverse events. Rare but significant are immune-related neuromuscular complications, impacting quality of life and prognosis. Using the TriNetX Global Health Research Network, 17,112 HNC patients treated with ICIs were analyzed for neuromuscular complications (inflammatory myopathies, myocarditis, myasthenia gravis, and neuropathies) identified via ICD-10 codes. Frequencies, timing, and survival probabilities were compared between patients with and without complications. The vast majority of the cohort received anti-PD-1 therapy. Neuromuscular complications occurred in 2.09% of patients (355/17,112), with 437 total diagnoses indicating multiple complications per patient. Inflammatory myopathies were most common (54.23%), followed by myocarditis (27.92%), neuropathy (9.38%), and myasthenia gravis (8.47%). Patients with Myasthenia gravis complications were older (76 vs. 69 years), while neuropathy was more frequent in men, and inflammatory myopathies skewed toward women. Complications typically emerged within the first few months of treatment, though inflammatory myopathies also developed after 12 months. Survival analysis showed no significant difference between patients with and without complications, except for myocarditis, which was associated with worse survival. With our very large cohort of patients treated with immune checkpoint inhibitors (ICIs), we can demonstrate that neuromuscular complications of ICI therapy are rare but clinically significant. Cardiac involvement, particularly myocarditis, carries the worst prognosis. Prompt recognition and multidisciplinary management by oncologists and neurologists are essential to optimize outcomes in affected patients.

## Introduction

The management of head and neck cancers (HNC), encompassing a wide array of malignancies with distinct etiologies, risk factors, and clinical outcomes, has seen significant advances in recent years^1,2^. Among the most promising innovations is the use of immune checkpoint inhibitors (ICIs), a class of immunotherapeutic agents targeting immune checkpoints such as programmed death-1 (PD-1), programmed death-ligand 1 (PD-L1), and cytotoxic T-lymphocyte-associated protein 4 (CTLA-4). These immune checkpoints, critical to the maintenance of self-tolerance and immune homeostasis, are frequently hijacked by tumor cells to evade immune surveillance. ICIs work by inhibiting these checkpoints, effectively restoring the immune system’s capacity to recognize and eliminate tumor cells. In patients with recurrent or metastatic HNC, ICIs have shown promising outcomes in terms of disease control and survival^3^.

However, with the increasing use of ICIs, new challenges have emerged regarding the management of immune-related adverse events (irAEs). ICIs are associated with a spectrum of irAEs that are distinct from the adverse events typically seen with cytotoxic chemotherapy or radiation therapy. These events arise from the immune system’s reactivation and can involve nearly any organ system, posing diagnostic and therapeutic challenges^4^.

Severe irAEs have been observed in up to 30% of individuals treated with CTLA-4 inhibitors, 10% of those treated with PD-1 inhibitors, and over 50% of those treated with combination therapy comprising CTLA-4 and PD-1/PD-L1 inhibitors^5^. Among these, neurological complications, although relatively rare, have proven to be particularly serious and difficult to manage. Serious neurological irAEs occur in approximately 1% to 2% of treated patients^6,7^.

Many different neurological irAEs affecting the central nervous system have been described. These include encephalitis, demyelination, myelopathy, vasculitis, cerebral aseptic meningitis and cranial neuropathies. In addition, irAEs affecting the peripheral nervous system (so-called PNS-irAEs) such as radiculitis, Guillain-Barré-like syndrome, peripheral neuropathy (including mononeuropathy, mononeuritis multiplex and polyneuropathy), myasthenic syndrome and myositis have also been described^8^.

Overall, irAEs are more common in the peripheral nervous system than in the central nervous system, and the incidence is likely to be underestimated. Muscle involvement (immune-related myopathy) is the most common PNS-irAE described and may also be associated with neuromuscular junction dysfunction. Immune-related myopathy (with or without neuromuscular junction involvement) also occurs in association with immune-related myocarditis, and this overlap syndrome is associated with significantly increased mortality^9^. Immune-related peripheral neuropathy most commonly occurs as polyradiculoneuropathy or cranial neuropathies^10^.

The severity of irAE often dictates whether ICI therapy may be continued. The most prevalent methodologies in such cases consist of the implementation of symptomatic and immunosuppressive therapy with glucocorticoids, immunoglobulins and/or plasmapheresis^11^ or a temporary discontinuation of the ICI^9^.

Therefore, the aim of this study is to examine the incidence of neuromuscular irAEs in patients receiving ICI therapy and the time of onset after initiation of therapy in a large cohort and to gain insight into the impact on long-term survival.

## Patients and methods

### Institutional review board statement

Ethical approval waiver: The requirement for ethical approval for this study was waived by institutional ethics committee of the Medical Faculty of Ulm University. Due to the retrospective nature of the study and the de-identification of the data, exclusively used from the TriNetX Global Health Research Network. This is because all Healthcare Organizations (HCOs) that contributed data to TriNetX acquired written informed consent from either the patients themselves or their legal guardians. This research was conducted in full accordance with the ethical standards of the institutional research committee as well as with the 1964 Helsinki declaration and its later amendments or comparable ethical standards. The TriNetX network contains data contributed by participating Healthcare Organizations. Each HCO affirms that it possesses all necessary rights, consents, approvals, and authority to supply data to TriNetX under a Business Associate Agreement (BAA). Importantly, these HCOs ensure that their identity remains anonymous as a data source, and the data they provide is exclusively used for research purposes. To further protect patient privacy, data shared through the TriNetX Platform undergoes attenuation, ensuring that it does not contain enough information to determine which specific HCO contributed patient information.

### Data Acquisition, allocation and matching

The TriNetX Global Health Research Network provides access to medical records from more than 120 healthcare organizations (HCOs) in over 30 countries in North and South America, EMEA and Asia-Pacific including over 250,000,000 patients with more than 5 years of clinical history enabling the collection and exchange of longitudinal clinical data^12^.

We enrolled patients who fulfilled the following inclusion criteria^1^: head and neck cancer (International Classification of Diseases (ICD)−10 codes: C00-C14, C30-C32 and C76) and^2^ immune-checkpoint-inhibitor therapy with PD-1-, PDL-1 and CTLA-4-inhibitor from the TriNetX network were used.

Exclusions criteria were^1^ follow-up data less than 5 years and^2^ patients with medical records older than 20 years^3^ previous diagnosis of one of the neuromuscular diseases investigated. There were no other specific selection criteria beyond the diagnosis and treatment type used for this study, to properly make use of real-word data. To be included, patients’ medical records had to cover at least five years (1825 days) of follow-up after visiting the HCO for an inpatient encounter. Medical records older than 20 years were excluded, to enhance the efficiency of processing and delivering outcomes analytics results, the TriNetX Global Health Research Network excludes patients from the results who met the criteria more than two decades ago. In most cohorts, only a minor fraction of patients falls into this category, as most of the data accessible through TriNetX pertains to patient encounters that have taken place within the past two decades.

The diagnosis of neuromuscular-irAEs was also previously defined using ICD-10 codes. For ICD-10 search for myositis: Drug-induced myopathy (G72.0), inflammatory and immune myopathies (G72.4), myopathy due to other toxic agents (G72.2), myositis (M60.9) and dermatopolymyositis (M33). For the complication myasthenia gravis, the ICD-10 codes myasthenia gravis (G70.0) and toxic myoneuronal disorders (G70.1) were selected. The complication myocarditis was analysed using acute myocarditis (I40) and Myocarditis, unspecified (I51.4). Neuritis as a complication of therapy was analysed using inflammatory polyneuropathy (G61). In the present study, myocarditis is categorized as a neuromuscular disease. The ICD-10 codes were selected by two neurologists who specialise in neuromuscular disorders. To truly identify only irAE and prevent overinterpretation due to concomitant tumour-related reactions, such as other non-inflammatory chemotherapy-induced polyneuropathies, critical illness neuropathies, paraneoplastic neuropathies and sarcopenia, the selection of ICD codes was limited. Time to neuromuscular irAE onset was calculated from the date of the first immune checkpoint inhibitor administration (index date) to the first newly documented diagnosis code of the respective neuromuscular complication.

Two groups were formed for survival analyses: Cohort I suffered no neuromuscular complications, whereas patients in cohort II suffered from neuromuscular complications after therapy.

As in previous published studies to exclude confounders, cohorts I and II were matched 1:1 with respect to age, sex, lymph node metastases, nicotine dependence, alcohol dependence and body mass index (BMI). In addition, matching via lymph node metastases also allows comparison of patients with and without advanced and metastatic states. Other confounders were not considered in this study. This made it possible to get as close as possible to randomized study conditions^13,14^.

### Data analysis

Descriptive statistics were used to describe baseline patient and surgery characteristics. All categorical variables were expressed as absolute values (n) and relative incidence (%). Patient age was presented with the mean value and standard deviation. A multivariable analysis was performed to find associations between gender and 5-year overall survival. The outcome was defined as “death” within a period of 5 years after the start of therapy. We focused on overall-survival, since progression is not typically recorded in structured electronic health record (EHR), which is the source of most of the data provided by Healthcare Organizations to TriNetX Kaplan-Meier analysis, Cox proportional hazards regression, and calculation of risk ratio (RR), odds ratio (OR), and hazard ratio (HR) were performed. Data analysis was limited to a period of 5 years after the first HNC diagnosis, as patients are regarded as healed in case of absence/no recurrence of HNC or metastases within the defined period. Statistical analysis was performed using the log-rank test with a *p* ≤ 0.05 as statistically significant. For 1:1 matching, the propensity score-matching algorithm was used, where logistic regression is used to calculate a propensity score for each patient in the group. This can range from 0 to 1 and indicated the predicted probability that a patient was in cohort I or II given the patients covariates. The greedy nearest-neighbor matching algorithm with a caliper of 0.1 pooled standard deviation was used. The caliper setting within TriNetX has a fixed value of 0.1.

## Results

### Baseline demographic characteristics and therapy regimen

A total of 537,457 patients with head and neck tumours were examined in the study. Of the total number of patients, 17,112 (3.18%) received immunotherapy comprising an immune checkpoint inhibitor.

The mean age of patients receiving an immunotherapy comprising an immune checkpoint inhibitor was 69 years. Of the patients, 29.86% were female. The majority of patients (68.85%) identified as “white”.

In the cohort of 17,112 patients undergoing immunotherapy, 355 individuals (2.07%) experienced 437 instances of neuromuscular irAEs over the course of the disease. The mean age of the 355 patients with neuromuscular complications was 69 years. The male to female ratio was 60.57% to 21.97%, respectively. The most common self-reported race was “white,” with a prevalence of 74.08%.

The mean age of patients without any neuromuscular complications was 69 years. The gender distribution of the patient cohort and the most common self-reported race was comparable to the total patient cohort (see Table 1).

**Table 1:** Demographics of the patient cohort with and without neuromuscular immune-related adverse events (irAE).

	All Patients receiving ICI	Patients with neuromuscular irAE	Patients without neuromuscular irAE
**Number of Patients (n)**	17,112	355	16,757
**Age (in years**,** mean)** [Min, Max, STD]	69 [4,90,13]	69 [15,90,13]	69 [4,90,13]
**Gender (in %)** Female Male Unknown	29.86 67.22 2.92	33.80 60.57 5.63	29.76 67.39 2.85
**Race (in %)** White Asian Black or African Other or Unknown Race	68.85 7.01 6.80 17,34	74.08 6.19 5.91 13.82	68.80 7.01 6.81 17.38

The most frequently utilised ICIs in the patient cohort were the PD-1 inhibitors (nivolumab, pembrolizumab, and cemiplimab), accounting for 15,840 (92.99%) of cases. Table 2 illustrates that 10,841 patients (68.44%) received pembrolizumab, the most frequently used ICI (see Table 2). No patient received concomitant ICI therapy.

**Table 2:** Overview of the used immune checkpoint inhibitor therapy in the cohort, *n* = 17,112.

	Drug	Total Number (%)
**PD-1 Inhibitors**	Pembrolizumab	10,841 (68.44%)
	Nivolumab	4921 (31.07%)
	Cemiplimab	78 (0.49%)
**Total Number**	15.840	
**PDL-1 Inhibitors**	Atezolimumab	615 (51.68%)
	Durvalumab	465 (39.07%)
	Avelumab	110 (9.24%)
**Total Number**	1.190	
**CTLA-4 Inhibitors**	Ipilimumab	1186 (100%)
**Total Number**	1.186	

### Incidence of neuromuscular immune-related adverse events in immune checkpoint therapy

In the cohort of 17,112 patients analysed, a total of 2.07% (*n* = 355) experienced neuromuscular irAEs during ICI therapy (Table 3). A total of 437 detectable neuromuscular diagnoses occurred in these patients, meaning that patients could be affected by more than one of the diagnoses analysed.

The most common complication was myopathy with 237 cases (54.23%). Looking more closely at the myopathies in terms of ICD-10 diagnoses, the most common myopathy was diagnosed as ‘myositis’. The second most common diagnosis was myocarditis. This was followed by neuropathies, which accounted for 9.38% of neuromuscular diagnoses. Myasthenia gravis was the rarest diagnosis (8.47% of all diagnoses).

For PDL-1 inhibitors and CTLA-4 inhibitors, the data collected for all neuromuscular irAEs was too small to calculate with sufficient accuracy.

**Table 3:** Total numbers of neuromuscular immune-related adverse events (irAEs) in patients treated with ICI (A value < 10 means, that the total number is too small in the TriNetX register to give a valid number of affected patients).

Neuromuscular irAE	Total Number (%)	PD-1 inhibitors	PDL-1 inhibitors	CTLA-4 inhibitors
Inflammatory Myopathies	237 (54.23%)	216 (52.81%)	< 10	< 10
Myositis	146 (33.41%)	130 (31.78%)	< 10	< 10
Drug induced myopathy	57 (13.04%)	53 (12.96%)	< 10	< 10
Dermatopolymyositis	23 (5.26%)	22 (5.38%)	< 10	< 10
Inflammatory and immune myopathies	11 (2.52%)	11 (2.69%)	< 10	< 10
Myopathy due to other toxic agents	< 10	< 10	< 10	< 10
**Myocarditis**	**122 (27.92%)**	**117 (28.61)**	**< 10**	**< 10**
Myocarditis	70 (16.02%)	67 (16.38%)	< 10	< 10
Acute myocarditis	52 (11.90%)	50 (12.23%)	< 10	< 10
**Neuropathies**	**41 (9.38%)**	**40 (9.78)**	**< 10**	**< 10**
Inflammatory Polyneuropathy	41 (9.38%)	40 (9.78%)	< 10	< 10
**Myasthenia gravis**	**37 (8.47%)**	**36 (8.80%)**	**< 10**	**< 10**
Myasthenia gravis	37 (8.47%)	36 (8.80%)	< 10	< 10
Toxic myoneural disorders	< 10	< 10	< 10	< 10

### Comparison of demographic characteristics for different neuromuscular immune-related adverse events

#### Inflammatory myopathies

The mean age of patients with neuromuscular immune-related complications in muscles was 69 years 39.29% of these patients were female. The most common racial group was “white” (74.10%). The mean age of patients without myopathic complications was 69 years. 29.56% of these patients were female. The most common self-reported race was “white,” with a prevalence of 68.70%.

#### Myocarditis

The mean age of patients with myocarditis complications was 69 years. 24.13% of these patients were female. The most common racial group was “white” (72.41%). The mean age of patients without myocarditis complications was 69 years. 29.72% of these patients were female. The most common self-reported race was “white,” representing 68.76% of the sample.

#### Myasthenia Gravis

The mean age of patients with myasthenic complications was 76 years. 28.95% of these patients were female. The most common racial group was “white” (78.94%). The mean age of patients without myasthenic complications was 69 years. 29.69% of these patients were female. The most common self-reported race was “white,” with a prevalence of 68.75%.

#### Neuropathies

The mean age of patients with neuropathic complications was 69 years. 23.80% of these patients were female. The most common racial group was “white” (73.80%). The mean age of patients without neuropathic complications was 69 years. 29.71% of these patients were female. The most common self-reported race was “white,” with 68.76% of the sample identifying as such.

### Occurrence of neuromuscular immune-related adverse events with PD-1 inhibitors over time

**Fig. 1:**
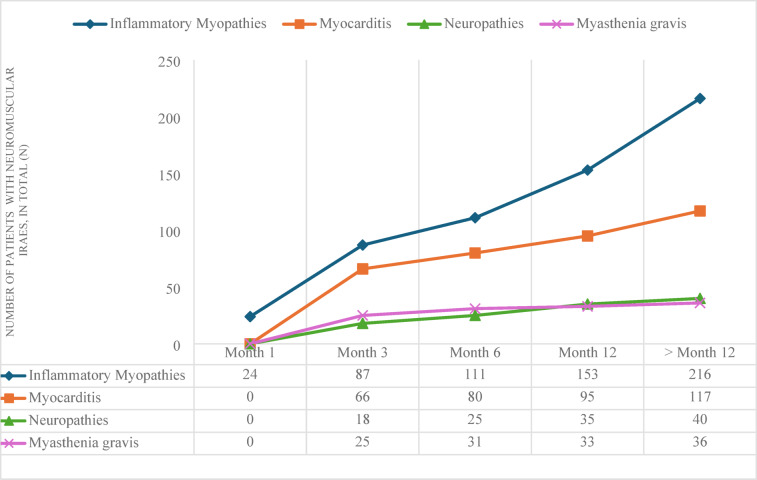
Occurrence of neuromuscular immune-related adverse events (irAEs) in patients treated with PD-1 inhibitors depending on time. Frequencies of diagnoses inflammatory myopathies, myocarditis, neuropathies or myasthenia gravis at the time points 1 month, 3 months, 6 months, 12 months and > 12 months.

A comprehensive analysis of **all PD-1 inhibitors** revealed a striking increase in the incidence of inflammatory myopathies over time, with a notable rise from 24 cases at one month to 216 cases after 12 months. Similarly, the incidence of myocarditis exhibits an upward trajectory, albeit at a slower rate. It increases from 66 cases after 3 months to 117 cases beyond 12 months. The progression of inflammatory polyneuropathy and myasthenia gravis was relatively stable, with slight increases in prevalence over time, resulting in 40 and 36 cases, respectively. Myocarditis, myasthenia gravis and inflammatory neuropathies were not detectable in the first month (Fig. 1).

**Fig. 2:**
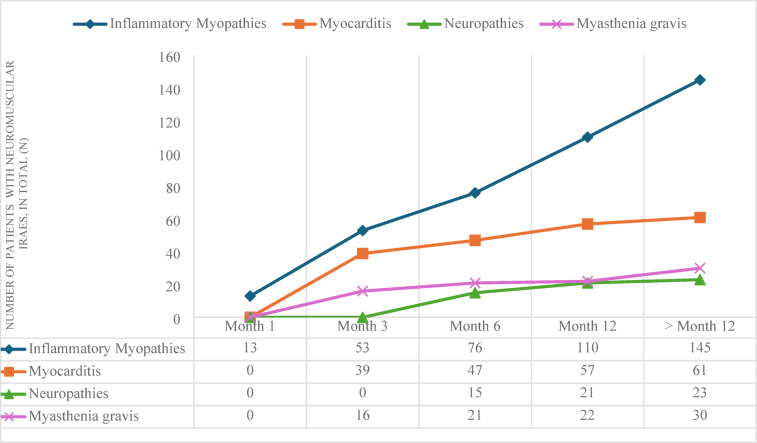
Occurrence of neuromuscular immune-related adverse events (irAEs) in patients treated with pembrolizumab depending on time. Frequencies of diagnoses inflammatory myopathies, myocarditis, neuropathies or myasthenia gravis at the time points1 month, 3 months, 6 months, 12 months and > 12 months.

The incidence of inflammatory myopathies in patients undergoing **pembrolizumab** treatment was observed to increase in a linear and continuous manner over time. At the initial observation period of one month, the incidence was 13 cases, while at the final observation period of 12 months, it reached 145 cases. The incidence of myocarditis showed a more gradual increase, starting from 39 cases at month 3 and growing to 61 cases beyond 12 months. Neuropathies occurred not before 3 months after the treatment with Pembrolizumab and increased slowly to 23 cases. A similar trend is observed in the prevalence of myasthenia gravis, which increases from 16 cases at 1 month to 30 cases beyond 12 months. (Fig. 2).

**Fig. 3:**
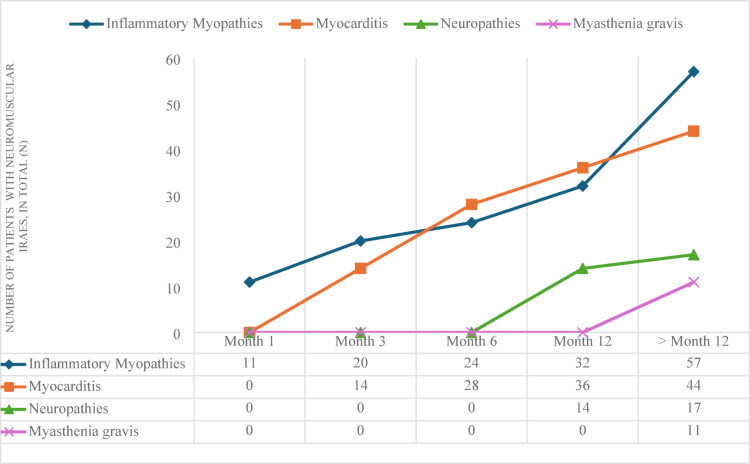
Occurrence of neuromuscular immune-related adverse events (irAEs) in patients treated with nivolumab depending on time. Frequencies of diagnoses inflammatory myopathies, myocarditis, neuropathies or myasthenia gravis at the time points: 1 month, 3 months, 6 months, 12 months and > 12 months.

In patients undergoing treatment with **nivolumab**, there has been a notable increase in the incidence of myositis over time. The number of cases increased from 11 at the initial time point to 57 in total. Similarly, the prevalence of myocarditis demonstrates a gradual increase, with 14 cases observed at the initial time point and 44 cases at the final time point. Neuropathies and myasthenia gravis occur predominantly at or beyond 12 months (Fig. 3). Due to the limited number of cases a detailed analyses of neuromuscular complications under a treatment with Cemiplimab over the time is not possible.

### Survival analysis

#### Survival analysis in all neuromuscular immune-related adverse events

Due to the propensity score matching performed for a 1:1 matching of patient cases per cohort, the following numbers of neuromuscular complications are different from the numbers shown in 3.1, which are not subject to propensity score matching, as patient cases that could not be matched 1:1 were not included in the following analyses.

##### Risk analysis

The risk of death was compared between patients without (Cohort 1, *n* = 349) and with neuromuscular complications (Cohort 2, *n* = 349). In Cohort 1, 167 patients (47.9%) experienced the outcome, compared to 182 patients (52.1%) in Cohort 2. The risk difference was − 0.043 (95% CI: −0.117 to 0.031), indicating a slightly lower risk in Cohort 1, though not statistically significant (z = −1.136, *p* = 0.256). The risk ratio (0.918; 95% CI: 0.791 to 1.065) and odds ratio (0.842; 95% CI: 0.626 to 1.133) similarly showed no significant risk reduction.

##### Kaplan-Meier survival analysis

**Fig. 4:**
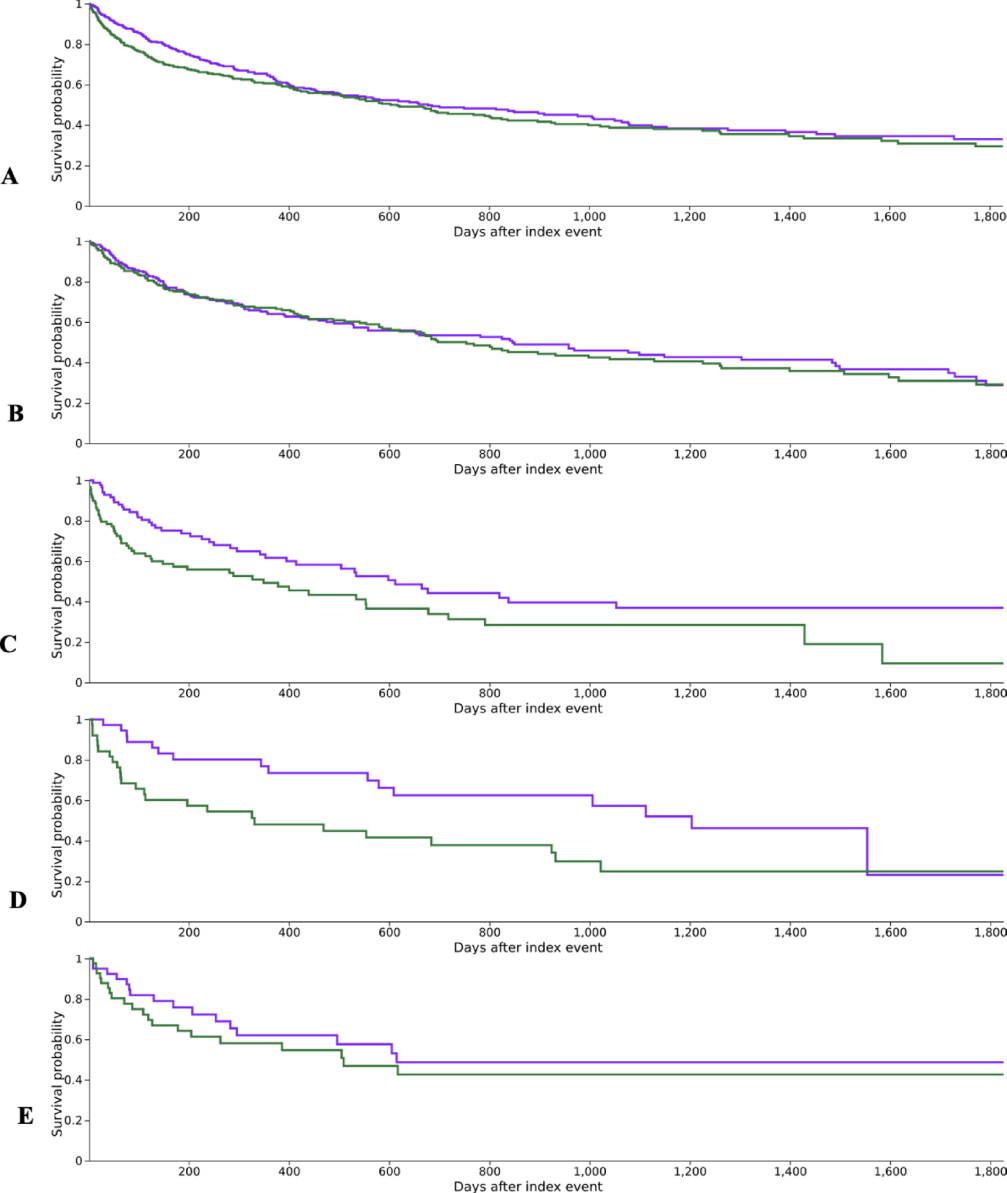
Survival analysis of patients with neuromuscular immune-related adverse events in comparison to patients without neuromuscular complications. **A**: Kaplan-Meier survival analysis of patients with any neuromuscular complication (Cohort 2, green) in comparison to patients with no neuromuscular complication (Cohort 1, violet). **B**: Kaplan-Meier survival analysis of patients with **inflammatory myopathies** (Cohort 2, green) in comparison to patients with no inflammatory myopathies (Cohort 1, violet). **C**: Kaplan-Meier survival analysis of patients with **myocarditis** (Cohort 2, green) in comparison to patients with no myocarditis (Cohort 1, violet). **D**: Kaplan-Meier survival analysis of patients with **myasthenia gravis** (Cohort 2, green) in comparison to patients with no myasthenia gravis (Cohort 1, violet). **E**: Kaplan-Meier survival analysis of patients with **neuropathies** (Cohort 2, green) in comparison to patients with no neuropathies (Cohort 1, violet).

Median survival was 678 days in Cohort 1 (33.02% survival probability) versus 617 days in Cohort 2 (29.49% survival probability). A log-rank test showed no significant difference between survival curves (χ² = 1.735, *p* = 0.188). The hazard ratio was 0.868 (95% CI: 0.704 to 1.071) with a χ² value of 3.362 and a marginal p-value of 0.067, suggesting a trend toward lower hazard in Cohort 1 without reaching statistical significance (Fig. 4A).

##### Mean number of instances

Patients in Cohort 1 had a higher mean outcome count (1.174 ± 0.380) compared to Cohort 2 (1.099 ± 0.299). This difference was statistically significant (t = 2.050, df = 347, *p* = 0.041).

#### Survival analysis in inflammatory myopathies

##### Risk analysis

The risk of death was similar between patients without myopathies (Cohort 1, *n* = 223) and those with myopathies (Cohort 2, *n* = 223). In Cohort 1, 47.1% (105 patients) experienced the outcome, compared to 50.2% (112 patients) in Cohort 2. The risk difference was − 0.031 (95% CI: −0.124 to 0.061), with no statistical significance (z = −0.663, *p* = 0.507). The risk ratio (0.938; 95% CI: 0.775 to 1.135) and odds ratio (0.882; 95% CI: 0.608 to 1.279) also indicated minimal differences between the cohorts.

##### Kaplan-Meier survival analysis

Kaplan-Meier analysis showed similar survival outcomes. Median survival was 846 days in Cohort 1 (28.71% survival probability) and 754 days in Cohort 2 (29.11% survival probability). The log-rank test (χ² = 0.133, *p* = 0.715) and hazard ratio (0.952; 95% CI: 0.729 to 1.242) showed no significant differences in survival or hazard between cohorts. The χ² test result (0.072, *p* = 0.789) further confirmed no statistical significance (see Fig. 4B).

##### Mean number of instances

The mean number of outcome instances was slightly lower in Cohort 1 (1.038 ± 0.192) compared to Cohort 2 (1.089 ± 0.286). This difference was not statistically significant (t = −1.535, df = 215, *p* = 0.126).

#### Survival analysis in myocarditis

##### Risk analysis

The risk of death was higher in patients with myocarditis (Cohort 2, *n* = 88) compared to those without (Cohort 1, *n* = 88). In Cohort 2, 59.1% (52 patients) experienced the outcome versus 46.6% (41 patients) in Cohort 1. The risk difference was − 0.125 (95% CI: −0.271 to 0.021), indicating a lower risk in Cohort 1, though not statistically significant (z = −1.661, *p* = 0.097). The risk ratio (0.788; 95% CI: 0.594 to 1.047) and odds ratio (0.604; 95% CI: 0.333 to 1.097) suggested a trend toward reduced risk and odds in Cohort 1, but neither reached statistical significance.

##### Kaplan-Meier survival analysis

Kaplan-Meier analysis showed a median survival of 612 days in Cohort 1 (36.93% survival probability) versus 349 days in Cohort 2 (9.49% survival probability). The log-rank test (χ² = 5.915, *p* = 0.015) indicated a statistically significant difference in survival. The hazard ratio was 0.603 (95% CI: 0.400 to 0.911), suggesting a lower hazard in Cohort 1, though the χ² value (1.688, *p* = 0.194) was not statistically significant (Fig. 4C).

##### Mean number of instances

The mean number of outcome instances was slightly higher in Cohort 1 (1.122 ± 0.331) compared to Cohort 2 (1.058 ± 0.235), but the difference was not statistically significant (t = 1.093, df = 91, *p* = 0.277).

#### Survival analysis in myasthenia Gravis

##### Risk analysis

Patients with myasthenic complications (Cohort 2, *n* = 38) had a higher risk of death (65.8%; 25 patients) compared to those without (Cohort 1, *n* = 38; 42.1%; 16 patients). The risk difference was − 0.237 (95% CI: −0.455 to −0.019), indicating a significantly lower risk in Cohort 1 (z = −2.071, *p* = 0.038). The risk ratio was 0.640 (95% CI: 0.413 to 0.991), and the odds ratio was 0.378 (95% CI: 0.149 to 0.958), both supporting a significantly reduced risk for Cohort 1.

##### Kaplan-Meier survival analysis

Kaplan-Meier analysis showed a median survival of 1204 days in Cohort 1 (23.14% survival probability) versus 331 days in Cohort 2 (24.85% survival probability). The log-rank test (χ² = 5.537, *p* = 0.019) indicated a significant difference in survival curves. The hazard ratio was 0.477 (95% CI: 0.254 to 0.896), suggesting a lower hazard in Cohort 1. However, the χ² test result (3.421, *p* = 0.064) was not statistically significant.

##### Mean number of instances

Cohort 1 had a slightly higher mean outcome count (1.313 ± 0.479) compared to Cohort 2 (1.080 ± 0.277). This difference approached statistical significance (t = 1.974, df = 39, *p* = 0.055).

#### Survival analysis in neuropathies

##### Risk analysis

Patients with neuropathies (Cohort 2, *n* = 42) had a slightly higher risk of death (47.6%; 20 patients) compared to those without neuropathies (Cohort 1, *n* = 42; 38.1%; 16 patients). The risk difference was − 0.095 (95% CI: −0.306 to 0.115), indicating no significant difference in risk (z = −0.882, *p* = 0.378). The risk ratio (0.800; 95% CI: 0.486 to 1.318) and odds ratio (0.677; 95% CI: 0.284 to 1.614) also showed no substantial differences between the cohorts.

##### Kaplan-Meier survival analysis

Median survival was 615 days in Cohort 1 (48.76% survival probability) versus 509 days in Cohort 2 (42.65% survival probability). The log-rank test (χ² = 0.613, *p* = 0.434) and hazard ratio (0.770; 95% CI: 0.399 to 1.487) showed no significant differences in survival or hazard. The χ² test result (0.515, *p* = 0.473) confirmed this lack of significance (see Fig. 4E).

##### Mean number of instances

Cohort 1 had a slightly lower mean outcome count (1.125 ± 0.342) compared to Cohort 2 (1.250 ± 0.444), but the difference was not statistically significant (t = −0.927, df = 34, *p* = 0.361).

## Discussion

### Frequency of neuromuscular IrAEs

The incidence of neuromuscular irAEs in the analysed cohort was 2.07%. Myopathies were the most common, accounting for 54.23% of neuromuscular irAEs. This was followed by myocarditis with a total of 27.92%. Peripheral neuropathy is much less common in the cohort analysed (9.38%). Myasthenia gravis was the least common (8.47%).

A total of 437 diagnoses were identified in the 355 patients affected by at least one neuromuscular irAE, suggesting additional co-occurrence of multiple neuromuscular irAEs or possible double coding of neuromuscular complications.

Patients suffered from neuromuscular irAE are of the same age as the total cohort. Patients with the complication myasthenia gravis are older than patients with the other neuromuscular irAEs as well as those without neuromuscular irAES (MG = 76 years, vs. neuropathy = 69 years, vs. inflammatory myopathy = 69 years). There is a slight emphasis on the female gender to the detriment of side effects (no neuromuscular irAE = 29.8% vs. neuromuscular irAE = 33.8% female) as well as a slight emphasis on the white race. Regarding the gender differences between the different neuromuscular irAEs, it is noticeable that on average more men (76%) are affected by neuropathy, whereas the complication of myositis has the highest proportion of women.

With the exception of extensive meta-analyses of case series and case reports, this survey of neuromuscular irAEs in a cohort of Head and Neck Cancer patients treated with ICI is the largest known to us. National studies from Spain (21 hospitals between January 2018 and February 2023) and Italy (7 hospitals between January 2016 and October 2023) reported 12 and 48 cases of neuromuscular irAEs, respectively, among 64 and 66 patients with neurological irAEs) However, these studies are limited by the lack of a denominator for the incidence of neuromuscular irAEs in the broader population of cancer patients. In our study, the incidence of neuromuscular complications is comparable to that observed in much smaller cohorts, where the incidence was described as 2.17% (out of 920 patients treated, 20 patients developed neuromuscular irAEs)^15^. The frequency of 2% as a value for neuromuscular irAEs can therefore be assumed.

In addition, discrepancies in the incidence of immune-related neurotoxicity have been observed in previous studies between the various immune checkpoint inhibitors. For instance, patients treated with ipilimumab exhibited neurological irAE in 1 to 1.6% of cases, while patients treated with anti PD 1 agents (nivolumab + pembrolizumab) exhibited irAE in 3 to 3.2% of cases. Furthermore, combination therapy involving several ICIs resulted in an even higher frequency of neurological irAEs, reaching up to 10%^16^. The study was not sufficiently robust to differentiate between the various ICIs in terms of frequency, as the majority of patients (*n* = 15,840) were treated with PD-1 inhibitors.

The distribution of the frequencies of the different neuromuscular irAEs, as found in the present study, has also been confirmed in other studies. A large meta-analysis also shows that the most common neurological irAE is myositis (approximately one third of all neurological irAEs), followed by Guillain-Barré syndrome and myasthenia gravis^17^. The findings of other smaller cohort studies also indicate myositis as the most common neuromuscular irAE. Additionally, cases of myocarditis have been documented^18^. The overall frequency of neuropathies is similar to the frequency observed in previous surveys. It is described as the third most common complication associated with PD-1, as in our study^19^.

As most patient case reports and large case series detail complications arising from neuromuscular irAE, further investigation is required to determine whether there is a gender prevalence, and whether different races or age groups are more vulnerable. For instance, age has been identified as a significant risk factor for the development of neuropathies, while women appear to be more likely to be screened for complications due to myositis.

### Occurrence of neuromuscular complications with PD-1 inhibitors over time

Regarding the PD-1 inhibitors and the respective incidence of neuromuscular irAEs, both the overall analysis and the individual analysis of the different PD-1 inhibitors show that myositis is detected earliest of all entities. The incidence of myositis continues to increase over the course of treatment, leading to additional new diagnoses. Myocarditis and myasthenia gravis are not detectable in the first month with any of the PD-1 inhibitors in our cohort, but are clearly present by the third month, with only a small increase in new diagnoses. The occurrence of neuropathy is the latest complication, for all PD-1 inhibitors analysed.

The exact onset of disease cannot be determined within the scope of the study design, but examination of the real-world data is consistent with the latencies described to date. This shows that myasthenia gravis and myositis have an overall shorter latency compared to neuropathies. For neuromuscular irAE, the mean latency from first ICI administration is reported to be 8.8 weeks on average^15^. However, there is also more variation in the occurrence of side effects, which can range from the first week to the 115th week^18^. The variation in the course of neuromuscular complications of ICI therapy is potentially influenced by factors such as fulminant, chronic, monophasic, or multiphasic disease courses, comedications, and concomitant underlying conditions^20^.

### Course of disease and survival

A significantly worse survival of patients with neuromuscular irAEs in HNC compared to patients without neuromuscular irAEs could not be demonstrated in the present study, but observation of the Kaplan-Meier curves shows a more rapid decline in survival at the onset of the disease in myasthenia gravis and myocarditis.

Overall, studies to date have described an increased mortality rate in myasthenia gravis, myositis and myocarditis compared to neuropathies due to ICI therapy. Myasthenia gravis mortality in connection with ICI therapy has been described as 10.9% and in combination with myocarditis and myositis as high as 34.5%^15^. The simultaneous occurrence of myocarditis with other neuromuscular complications, such as myasthenia gravis and myositis, is an additional risk factor for a poor outcome^21^. It is therefore indicated to rule out myocarditic involvement during ICI therapy as well as during the development of neuromuscular complications. It has been described that the symptoms and presentation can be challenging. Symptoms range from chest pain, dyspnoea, syncope, palpitations, pulmonary oedema to cardiogenic shock. Myocarditis may also present initially with arrhythmias and delayed diagnosis^22^.

Overall, neuromuscular irAEs are rare, but due to the severe symptom burden and need for alternative therapy, it is important that they are recognised and treated early as there is an increased risk of mortality and morbidity. The symptoms of the disease are generally challenging and difficult to recognise in patients with tumours, as typical symptoms include muscle weakness, sarcopenia, fatigue, malaise, dysphagia, shortness of breath and exercise intolerance, which are also common in patients with tumours. Close collaboration with oncology and neurology specialists is essential for optimal management, treatment and follow-up of this serious complication.

### Strengths and limitations

The real-world data analysis demonstrates several strengths, including a large patient cohort, a multi-institutional approach, and the use of propensity score matching to reduce the impact of confounding factors. It is notable that multi-institutional outcomes are underrepresented in the existing literature. The study performed matching based on age and common risk factors, such as smoking and alcohol consumption, using data from the TriNetX database. However, the database’s limitations precluded the investigation of HPV status and other histopathological parameters, such as UICC stage, lymph node metastasis, or extracapsular spread. TNM staging data was available only for patients whose records originated from cancer registries, and for other sources, TNM data was stored in free-text format, requiring extraction through natural language processing (NLP). The implementation of NLP is both financially and resource-intensively, and there is a need for robust data quality control. Additionally, reliance on ICD-10 codes prevented the extraction of information about tumour stage and specific pathohistological features.

It is imperative to acknowledge the limitations of the study. Firstly, the cause of death data, which is crucial for distinguishing cancer-related mortality, was not available in the TriNetX database. Secondly, the real-world data was sourced from healthcare organisations across Europe, the Middle East, Africa, Asia, North America, and South America. This global, multi-centre analysis did not account for national or international variations in the treatment and epidemiology of oral cancer.

As a retrospective and descriptive study, the integrity of the data was not within the authors’ control, and patients may have been lost to follow-up due to transferring care to healthcare organisations outside the TriNetX network. In addition, the lack of access to raw data limited the ability to conduct additional or separate analyses, and these limitations constrain the applicability of the findings for guiding clinical practice.

Diagnoses were recorded using ICD-10 coding. For the diagnosis search, methodologically typical ICD-10 codes were used, which were carefully selected in advance. However, it cannot be excluded that ICI complications were coded differently. Since the ICD-10 codes were defined very restrictive, it is possible that the actual frequency of neuromuscular irAE was underestimated. The codes for neuropathies in particular were purposely defined restrictive in order to reduce overlap with other therapy- and tumour-related neuropathies.

It cannot be excluded that patients could have developed MG or inflammatory myopathy after tumour diagnosis and ICI independently of therapy. However, this is unlikely due to the side effect profile of ICI therapy. Myositis is also associated with tumours. A reliable differentiation is not possible due to the methodology.

Furthermore, there were no data and clinical features concerning the type of neuropathies, this limited the ability to match comparative groups in terms of disease severity.

Moreover, the clinical distinction between ICI-related myositis and myasthenia gravis is often challenging, and overlap syndromes are increasingly recognized. As our analyses are based on routine ICD-coded diagnoses, some patients may have been preferentially coded as myasthenia gravis rather than myositis (or vice versa), potentially inflating the apparent myasthenia caseload at the expense of myositis. This diagnostic and coding uncertainty should be considered when interpreting the entity-specific incidence estimates.

The findings need to be interpreted cautiously regarding the limitations discussed above.

## Conclusion

ICIs represent a revolution in cancer therapy, but due to their mechanism of action they have new side effects, including immune-related adverse events of the peripheral nervous system, including myasthenia gravis, myositis and neuropathy, also in combination with myocarditis. Although the incidence is low, severe disease can occur shortly or months after treatment and is difficult to diagnose in patients with tumours. A combination of these conditions may also occur.

As the use of ICIs continues to increase, ongoing research and real-world data from global registries, as present here, are essential to better understand ICI-associated neuromuscular irAEs and myocarditis, prevent them, and treat them promptly for the benefit of cancer patients.

Large prospective cohorts and real-world studies of other tumour types are also needed to investigate risk factors for complications and other aspects of side effects. Interdisciplinary collaboration between oncologists and neurologists is essential for neuromuscular irAEs.

## Data Availability

The data supporting the findings of this study are available from the corresponding author upon reasonable request or directly from the TriNetX Global Health Research Network, subject to applicable data use agreements.

## Abbreviations

BAA: Business Associate Agreement
CTLA-4: Cytotoxic T-lymphocyte-associated protein 4
HCOs: Healthcare Organizations
HIPAA: Health Insurance Portability and Accountability Act
HNC: Head and neck cancers
HR: Hazard ratio
ICD: International Classification of Diseases
ICI: Immune checkpoint inhibitors
IRB: Institutional Review Board
irAE: Immune-related adverse events
ISMS: Information Security Management System
MG: Myasthenia gravis
NLP: Natural language processing
OR: Odds ratio
PD-1: Programmed death-1
PD-L1: Programmed death-ligand 1
PNS: Peripheral nervous system
PNS-irAE: Peripheral nervous system immune-related adverse events
RR: Risk ratio
RWD: Real-world data
SD: Standard deviation
